# Supplementary material for: Effect of non-crop vegetation types on conservation biological control of pests in olive groves
Source: PeerJ. 2013 Jul 25;1:e116. doi: 10.7717/peerj.116 (PMC3728767; doi:10.7717/peerj.116)
Supplement: Appendix S1 [file peerj-01-116-s001.docx]

#######################################################

### LOADING PACKAGES AND DATA

#######################################################

library(likelihood)

library(spatstat)

library(maptools)

dat <- read.table("…/data.txt")

str(dat)

### 'dat' contains the following variables: (1) Response variable (in our case *E. olivina* and *P. oleae*); (2) Time: a continuous variable measured in Julian days; (3) Treatment: a categorical variable with two levels (ground cover and bare soil); (4) Year: a categorical variable with two levels (2010 and 2011)

#######################################################

### CALCULATING AND INCLUDING BIVARIATE KERNEL DENSITY FUNCTIONS OF VEGETATION

#######################################################

plots <- readShapePoints("…/.shp") # A point layer containing the location of plots within the orchard

Lwv <- readShapePoints("…/.shp") # A polygon layer containing the location of the points obtained by superimposing a grid of 1x1m grain over the patches of large woody vegetation.

Herb<- readShapePoints("…/.shp") # A polygon layer containing the location of the points obtained by superimposing a grid of 1x1m grain over the patches of large woody vegetation.

Swv <- readShapePoints("…/.shp") # A polygon layer containing the location of the points obtained by superimposing a grid of 1x1m grain over the patches of large woody vegetation.

coords.plots <- coordinates(plots)

coords.plots <- data.frame(coords.plots, Lwv=rep(NA, dim(coords.plots)[1]), Herb=rep(NA, dim(coords.plots)[1]), Swv=rep(NA, dim(coords.plots)[1]))

coords.Lwv <- coordinates(Lwv)

coords.Herb <- coordinates(Herb)

coords.Swv <- coordinates(Swv)

ppp.Lwv <- ppp(coords.Lwv[,1], coords.Lwv[,2], window=owin(range(coords.Lwv[,1]), range(coords.Lwv[,2])))

dens.Lwv <- density.ppp(ppp.Lwv, sigma=120)

coords.plots[,3] <- dens. Lwv [coords.plots[,1:2]]

ppp.Herb <- ppp(coords. Herb [,1], coords. Herb [,2], window=owin(range(coords.Lwv[,1]), range(coords.Lwv[,2])))

dens. Herb <- density.ppp(ppp. Herb, sigma=120)

coords.plots[,4] <- dens. Herb [coords.plots[,1:2]]

ppp.Swv <- ppp(coords.Swv[,1], coords.Swv[,2], window=owin(range(coords.Lwv[,1]), range(coords.Lwv[,2])))

dens.Swv <- density.ppp(ppp.Swv, sigma=120)

coords.plots[,5] <- dens.Swv[coords.plots[,1:2]]

dat$Lwv <- rep(coords.plots[,3], )

dat$Herb <- rep(coords.plots[,4], )

dat$Swv <- rep(coords.plots[,5], )

##########################################

####MODELLING

## In this work the basic Gaussian function (#1) was modified by including a set of new parameters (*dHerb, dLwv*, *dSwv*) that reflect the effect of herbaceous, large and small woody vegetation density on pest abundance (#4). The basic function was also change to account for differences in the estimated parameters across treatments [tr] (ground cover and bare soil; parameter *a* (#2) and *d*) and years [yr] (2010, 2011; parameters *a* (#3), *b*, *c* and *d*). The most complex model took into account the effects of ground cover, natural vegetation, the interaction between these two, and inter-annual variability in all the parameters (#5). Overall we tested 60 models with the possible combinations explained above.

###########################################

############################################

# 1. SIMPLE GAUSSIAN MODEL (NULL MODEL)

############################################

gausres <- function(a, b, c, X1) {

a*exp(-((X1-b)^2/2*c^2))

}

############################################

# 2. GAUSSIAN MODEL IN WHICH PARAMETER 'a' VARY AMONG TREATMENTS

############################################

gausres.tra <- function(a, b, c, tr, X1) {

a[tr]*exp(-((X1-b)^2/2*c^2))

}

############################################

# 3. GAUSSIAN MODEL IN WHICH PARAMETER 'a' VARY AMONG YEARS

############################################

gausres.yra <- function(a, b, c, yr, X1) {

a[yr]*exp(-((X1-b)^2/2*c^2))

}

############################################

# 4. GAUSSIAN MODEL WITH AN EFFECT OF VEGETATION

############################################

gausres.veg <- function(a, b, c, dLwv, dHerb, dSwv, X1, XLwv, XHerb, XSwv) {

(a+( dLwv * XLwv + dHerb * XHerb + dSwv * XSwv))*exp(-((X1-b)^2/2*c^2))

}

############################################

#5. GAUSSIAN MODEL WITH AN EFFECT OF VEGETATION IN WHICH PARAMETER 'a' AND "d" VARY AMONG TREATMENTS AND "a", "b" ,"c" AND “d” AMONG YEARS

############################################

gausres.veg.trad.yrabc <- function(a, b, c, dLwv, dHerb, dSwv, X1, XLwv, XHerb, XSwv, tr, yr) {

(a[tr][yr]+( dLwv [tr] [yr]* XLwv + dHerb [tr] [yr]* XHerb + dSwv [tr] [yr]* XSwv))*exp(-((X1-b[yr])^2/2*c[yr]^2))

}

###########################################

############################################

# MODEL INFERENCE

############################################

############################

# 1 Model

#############################

par<-list(a = max(dat$Response variable), b = 150, c=0.02)

var <- list(X1 = "Time")

par_lo<-list(a = 0.0000001, b = 0.000001, c=0.0000001)

par_hi<-list(a = 1000, b = 1000, c=1000)

var$x<-" Response variable "

var$lambda<-"predicted"

var$log<-TRUE

##now call the annealing algorithm, specifying the proper model

x11()

results.Response variable1<-anneal(gausres,par,var,dat,par_lo,par_hi,dpois," Response variable ", hessian = TRUE, max_iter=100000)

##now write results1 to a file

write_results(“results.Response variable1,…/results1.txt")

save.image(“…/analisis_Response variable.RData")

#############################

# 2 Model

#############################

par<-list(a = rep(max(dat$ Response variable),2), b = 150, c=0.02)

var <- list(X1 = "Time", tr="Treatment")

par_lo<-list(a = rep(0.0000001,2), b = 0.000001, c=0.0000001)

par_hi<-list(a = rep(1000,2), b = 1000, c=1000)

var$x<-" Response variable "

var$lambda<-"predicted"

var$log<-TRUE

##now call the annealing algorithm, specifying the proper model

x11()

results.Response variable2<-anneal(gausres.tra,par,var,dat,par_lo,par_hi,dpois,"Response variable", hessian = TRUE, max_iter=100000)

##now write results2 to a file

write_results(“results.Response variable2,…/results2.txt")

save.image(“…/analisis_Response variable.RData")

#############################

# 3 Model

#############################

par<-list(a = rep(max (dat$Response variable), 2), b = 150, c=0.02)

var <- list(X1 = "Time", yr=”Year")

par_lo<-list(a = rep(0.0000001,2), b = 0.000001, c=0.0000001)

par_hi<-list(a = rep(10000,2), b = 1000, c=1000)

var$x<-"Response variable"

var$lambda<-"predicted"

var$log<-TRUE

##now call the annealing algorithm, specifying the proper model

x11()

results.Response variable3<-anneal (gausres.yra,par,var,dat,par_lo,par_hi,dpois,"Response variable", hessian = TRUE, max_iter=100000)

##now write results3 to a file

write_results(“results.Response variable3,…/results3.txt")

save.image(“…/analisis_Response variable.RData")

#############################

# 4 Model

#############################

par<-list(a = max(dat$Response variable), b = 150, c=0.02, d2=100,d3=100,d4=100)

var <- list(X1 = "Time", XLwv = "Lwv", XHerb="Herb", XSwv="Swv")

par_lo<-list(a = 0.0000001, b = 0.000001, c=0.0000001, d2=-100000, d3=-100000,d4=-100000)

par_hi<-list(a = 1000, b = 1000, c=1000, d2=100000,d3=100000,d4=100000)

var$x<-"Response variable"

var$lambda<-"predicted"

var$log<-TRUE

##now call the annealing algorithm, specifying the proper model

x11()

results.Response variable4<-anneal(gausres.veg,par,var,dat,par_lo,par_hi,dpois,"Response variable", hessian = TRUE, max_iter=100000)

##now write results4 to a file

write_results(“results.Response variable4,…/results4.txt")

save.image(“…/analisis_Response variable.RData")

############################

# 5 Model

#############################

par<-list(a = rep(max(dat$Response variable),4), b = rep(150,2), c=rep(0.02,2), d2=rep(100,2), d3=rep(100,2), d4=rep(100,2))

var <- list(X1 = "Time", XLwv = "Lwv",XHerb="Herb", XSwv="Swv", tr="Treatment", yr="Year")

par_lo<-list(a = rep(0.0000001,4), b = rep(0.000001,2), c=rep(0.0000001,2), d2=rep(-100000,2), d3=rep(-100000,2), d4=rep(-100000,2))

par_hi<-list(a = rep(5000,4), b = rep(1000,2), c=rep(1000,2), d2=rep(100000,2), d3=rep(100000,2), d4=rep(100000,2))

var$x<-"Response variable"

var$lambda<-"predicted"

var$log<-TRUE

##now call the annealing algorithm, specifying the proper model

x11()

results.Response variable5<-anneal(gausres.veg.trad.yrabc,par,var,dat,par_lo,par_hi,dpois,"Response variable", hessian = TRUE, max_iter=100000)

##now write results5 to a file

write_results(“results.Response variable5,…/results5.txt")

save.image(“…/analisis_Response variable.RData")
